# Comparison of Investigations Following Inpatient and Outpatient Endoscopic Retrograde Cholangiopancreatography and Their Impact on Complication Rates and Length of Hospital Stay: A Retrospective Cohort Study

**DOI:** 10.7759/cureus.95878

**Published:** 2025-11-01

**Authors:** Owen Morris, Luc Nyunt

**Affiliations:** 1 Trauma and Orthopaedics, Wrexham Maelor Hospital, Wrexham, GBR; 2 General and Colorectal Surgery, Wythenshawe Hospital, Manchester, GBR

**Keywords:** cost reduction, endoscopic retrograde cholangiopancreatography (ercp), post-ercp complications, prolonged admission, routine venepuncture

## Abstract

Introduction

Endoscopic retrograde cholangiopancreatography (ERCP) is a therapeutic procedure for conditions affecting the biliary and pancreatic ductal systems. Complications include post-ERCP pancreatitis (PEP), cholangitis, and bleeding, which typically present within the first few hours post-procedure. This study aimed to determine if investigations following ERCP were indicated with respect to the European Society of Gastroenterology and Endoscopy (ESGE) guidelines and if there was variation in practice for inpatients versus outpatients.

Methods

Data were collected retrospectively from the endoscopy software Endobase (Olympus Corporation, Hamburg, Germany) for all patients who underwent an inpatient and outpatient ERCP over a six-month period between November 2022 and April 2023 at a district general hospital in Northwest England. Subsequent data post ERCP were collected from electronic patient records (EPR), including length of stay, reason for overnight admission, readmission within seven days, and mortality within seven days. Data were analysed using JASP 0.95.2 (JASP team, University of Amsterdam, Amsterdam, Netherlands (jasp-stats.org)) and compared against the ESGE guidelines as recommendations of best practice.

Results

During the six-month period, 134 patients underwent an ERCP, of which 69% (n=92) were inpatients and 31% (n=42) were outpatients. Out of the 92 inpatients, only 3.3% were discharged the same day compared to 95.2% of day cases ERCPs (p < 0.0001). In the inpatient cohort, 51% (n=45) were admitted for asymptomatic routine investigations (blood or observation) with no indication as per ESGE guidelines. None of the 45 patients admitted were diagnosed with an ERCP-related complication. This resulted in 63 additional bed days and 50 additional episodes of venepuncture. Out of the day case cohort (n=42), no patients were admitted for asymptomatic routine investigations. There was no significant difference in the overall complication rates (p=0.339) between day case and inpatient ERCP.

Conclusion

Our study supports current ESGE guidance that routine investigations following ERCP are not necessary in asymptomatic patients with no risk factors for complications. Over half of patients (51%) admitted following an inpatient ERCP could have been safely discharged the same day following a six-hour observation period without the need for further investigation.

## Introduction

Endoscopic retrograde cholangiopancreatography (ERCP) is a therapeutic procedure for conditions affecting the biliary and pancreatic ductal systems. It is often used to manage obstructive jaundice secondary to common bile duct stones and malignant biliary strictures [1]. ERCP procedures can be performed either as a day case or during an inpatient admission. ERCP is generally considered to be effective and safe. However, complications following ERCP include post-ERCP pancreatitis (PEP), cholangitis, bleeding, perforation, cholecystitis, cholangitis, and sedation-related complications such as hypoxaemia or hypotension [2]. 

The European Society of Gastrointestinal Endoscopy (ESGE) provides guidelines to help manage and identify possible complications that can develop following ERCP. Apart from sedation-related complications during an ERCP, the most frequent complication is PEP, which is defined by the ESGE as ‘new or worsening abdominal pain combined with greater than three times the normal value of amylase at more than 24 hours after ERCP and requirement of admission or prolongation of planned admission [2,3]. The reported incidence of PEP is between 3.5% and 9.7% [4, 5]. The reported mortality rates are low, between 0.1% and 0.7% [5].

Amylase has been shown to be elevated in up to 75% of patients following ERCP, whether symptomatic or not [5]. Abdominal pain is often the first presenting symptom of PEP. Hence, the ESGE guidance recommends that amylase should be tested if patients complain of abdominal pain post-procedure [2]. The British Society of Gastroenterology (BSG) guidelines recommend urgent review by a clinician if pain is not settling with analgesia, but state amylase testing is not always required, as it may be difficult to implement [6].

There are currently no specific guidelines for liver function tests (LFTs) by either the ESGE or BSG. It is not uncommon for LFTs to be asymptomatically deranged following ERCP, potentially due to a transient increase in biliary pressure [7]. Hence, deranged LFTs should not be used to determine complications in the absence of other symptoms, such as pyrexia or new or worsening abdominal pain, that would suggest complications such as cholangitis.

In over 90% of cases, post-ERCP complications are evident within six hours of the procedure [8]. In principle, investigations post ERCP are only indicated if symptomatic, according to ESGE guidance. The BSG guidance recommends that most patients will require a minimum observation period of four hours [6]. This forms the basis of the day case ERCP services, followed by a short period of observation on the endoscopy recovery unit prior to same-day discharge.

Clinicians within our gastroenterology department had noticed that day-case patients for an ERCP were more likely to be discharged the same day compared to those who were already admitted as inpatients. In addition, it appeared there were more investigations requested following inpatient ERCP compared to day cases. Current literature suggests similar complication rates for day case and inpatient ERCPs [9]. 

Given these variations in post-ERCP practice, we aimed to evaluate if there were differences between investigations requested following inpatient versus outpatient ERCP within a single district general hospital in Northwest England and if they were indicated with respect to ESGE guidelines. 

## Materials and methods

The lead ERCP operator had anecdotally noticed a difference in the length of admission and investigations requested for patients undergoing ERCP as an inpatient compared to as an outpatient. This formed the basis of developing a retrospective observational audit, which aimed to determine if investigations following ERCP were indicated with respect to the ESGE guidelines as recommendations of best practice. ESGE guidelines recommend that amylase should only be tested if patients complain of abdominal pain post-procedure [2]. Subsequently, we observed if there were any differences in practice when comparing the inpatient and outpatient cohorts.

The project team consisted of two Foundation Year 2 (FY-2) doctors, with monthly meetings with the consultant gastroenterologist who performed ERCP procedures and a general surgical consultant. Approval for the audit was obtained from the clinical audit department at Royal Bolton Hospital, Bolton, England (approval number: 4360). 

The primary objectives of the study were as follows: 1. Determine if investigations requested following ERCP were indicated with respect to ESGE guidelines; 2. Determine if there was a significant difference in non-indicated investigations requested between day case and inpatient ERCP.

The secondary objectives were: 1. Determine the impact that non-indicated investigations following ERCP had on patient length of stay and resources; 2. Identify if there is a significant difference in complication rates following ERCP between day cases and inpatients; 3. Determine if there was a significant difference in same-day discharge rates following ERCP between day cases and inpatients.

Data were collected retrospectively by a single FY-2 doctor from the electronic patient records (EPRs) used for ERCP procedures called Endobase (Olympus Corporation, Hamburg, Germany) for all patients who underwent an ERCP over a six-month period at a district general hospital in Northwest England between the 1^st^ November 2022 and 30^th^ April 2023. This was obtained by setting the procedure search filter to ERCP and the time filter to within the six-month time frame. The inclusion criterion was any ERCP performed within the six-month period. This resulted in a complete data set of 134 patients, of which 83 were female and 51 were male. No power calculation was performed, as a six-month period was chosen for the initial audit data collection to provide a sample size of at least 40 patients in each cohort that we believed would demonstrate results that could lead to implementation of change within the department. Exclusion criteria included incomplete procedure notes and incomplete electronic notes, of which there were none. 

The data collected from Endobase included date of procedure, sex of patient, date of birth, operator endoscopist, indication for ERCP, and whether the patient was a day case or an inpatient.

Subsequent data post ERCP were collected by the same FY-2 doctor from the EPR software Sunrise (Allscripts Healthcare Solutions, Chicago, IL, USA), which included length of stay, reason for overnight admission, re-admission within seven days, and mortality within seven days. This involved reviewing the electronic notes of each patient from the date of the ERCP to seven days post-discharge. Post-ERCP investigations were defined as next-day blood tests or admission for overnight observation.

Non-indicated investigations were defined as investigations performed on asymptomatic patients with no symptoms suggestive of a post-ERCP complication as per the ESGE guidelines. 

All patient data was anonymised, and only relevant patient data was accessed. Data was collected using Microsoft Excel (Microsoft Corp., Redmond, WA, USA). Tables and graphs were created using Microsoft Excel. The data were imported for further analysis, and Fisher's exact test was performed on the complication rates between inpatient and outpatient cohorts using an open-source, free-to-use software, JASP 0.95.2 (JASP team, University of Amsterdam, Amsterdam, Netherlands (jasp-stats.org)). 

The findings were presented at the Endoscopy Audit meeting at Royal Bolton Hospital.

## Results

In total, 134 patients underwent an ERCP during the six-month period; 69% (n=92) were inpatients, and 31% (n=42) were day case procedures. In the inpatient cohort, 58 were female (63%) and 34 male (37%), and in the day case cohort, 25 were female (60%) and 17 male (40%).

The most common indication for ERCP was suspected choledocholithiasis (78%), followed by cholangitis (7%), as outlined in Table 1. 

**Table 1:** Summary of indications for endoscopic retrograde cholangiopancreatography

Indications for ERCP	Day case (n=42)	Inpatient (n=92)
Suspected bile duct stone	31 (74%)	74 (80%)
Obstructive jaundice	4 (10%)	8 (9%)
Pancreatic mass	3 (7%)	0
Cholangitis	1 (2%)	7 (8%)
Bile duct leak	1 (2%)	2 (2%)
Hepatobiliary mass/cancer	1 (2%)	1 (2%)
Biliary stent removal	1 (2%)	0

**Figure 1:**
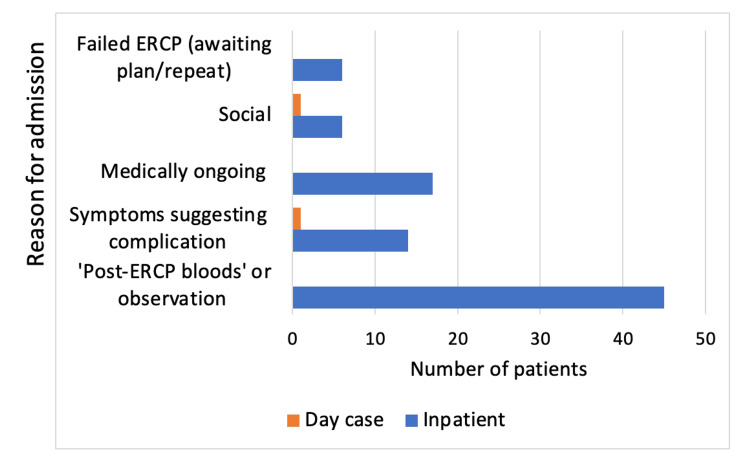
Reasons for prolonged admission following endoscopic retrograde cholangiopancreatography (ERCP)

Figure 1 shows the reasons for admission of the 88 inpatients post ERCP; 51% (n=45) were for post-ERCP routine investigations or observation, 19% (n=17) for ongoing medical management (IV antibiotics, awaiting scans, patient-controlled analgesia, or awaiting cholecystectomy), 16% (n=14) for symptoms suggesting complication, 7% (n=6) were due to failed ERCP awaiting a plan or repeat, and 7% (n=6) were for social reasons.

No patient admitted for asymptomatic routine investigations (post-ERCP blood tests including full blood count, LFTs, urea and electrolytes and amylase or observation) following inpatient ERCP had an ERCP-related complication diagnosed.

**Figure 2:**
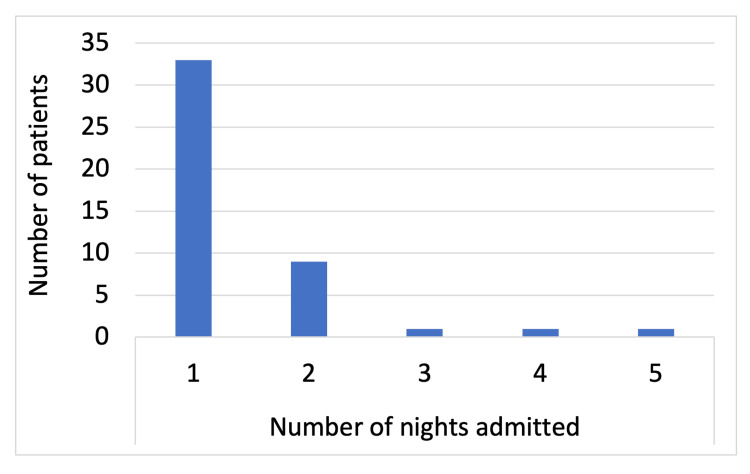
Number of nights admitted for blood tests/observations following an inpatient endoscopic retrograde cholangiopancreatography in asymptomatic patients

Figure 2 shows the resulting length of stay of patients who were admitted for asymptomatic routine investigations post inpatient ERCP, which was 73% (n=33) for one night, 20% (n=9) for two nights, and 2% (n=1) for three days, four days and five days, respectively, as shown in Figure 2. This totalled 63 additional nights, and a bed was occupied. In that time, there were a total of 50 additional blood tests.

Only two patients were admitted overnight after day case ERCP; no patients were admitted for asymptomatic investigations, and one was admitted for symptoms suggestive of complications (abdominal pain). The patient's admission resulted in an eight-night admission with five sets of blood being taken. The patient was subsequently diagnosed with a post-ERCP cholecystitis. One patient was admitted for social reasons. 

**Figure 3:**
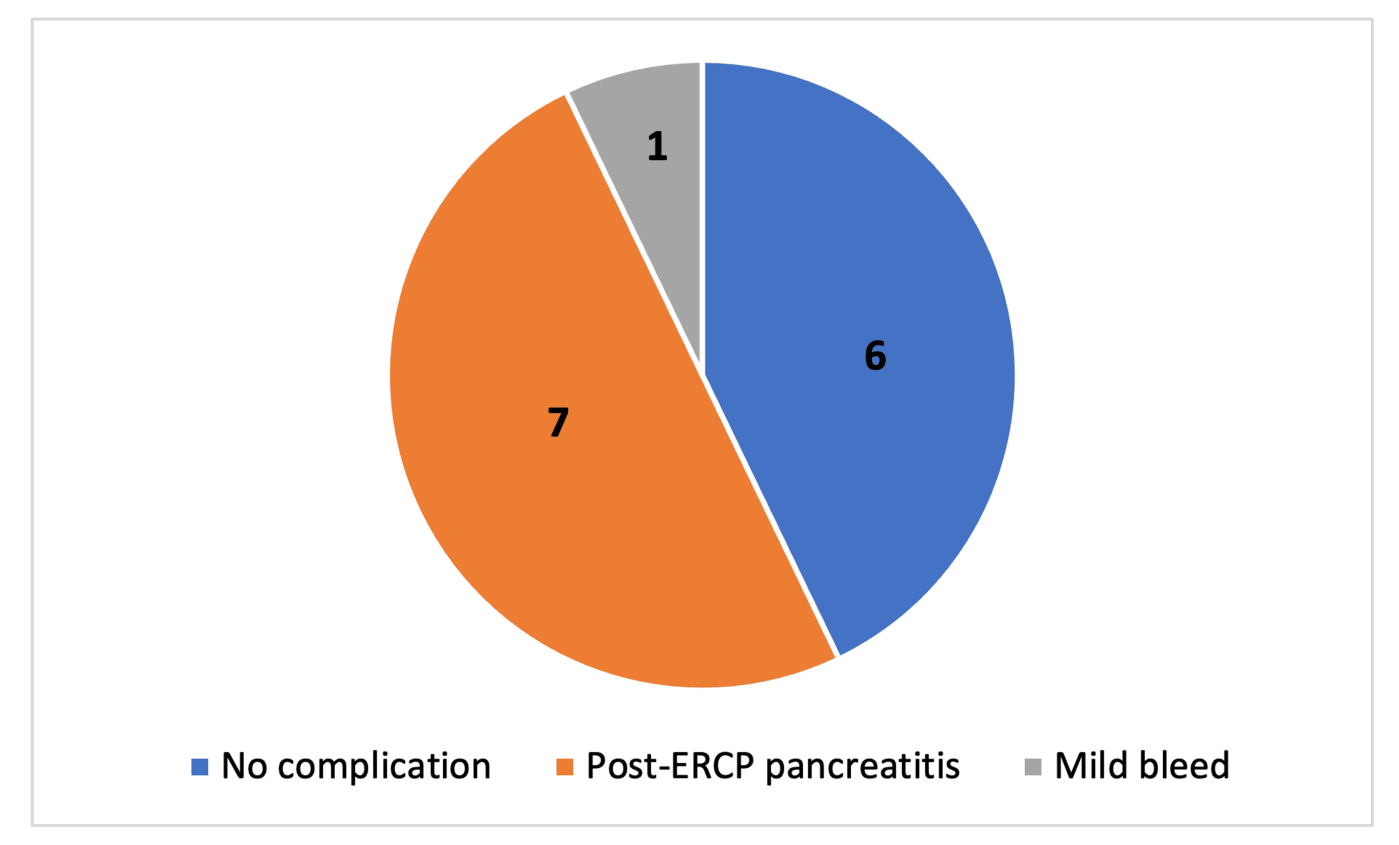
Distribution of complications associated with patients who had symptoms suggestive of complication following inpatient endoscopic retrograde cholangiopancreatography (ERCP)

In the inpatient ERCP cohort, 14 patients were admitted for symptoms suggestive of a complication, and eight were subsequently diagnosed with a complication. Seven patients were diagnosed with PEP and one with a mild post-ERCP bleed. Six patients had no complications diagnosed. This is shown in Figure 3. 

The overall complication rates for day cases and inpatients were not significantly different: 4.8% and 10.9%, respectively (p=0.339).

The incidence of PEP for day case and inpatient ERCP was 2.4% and 8.7%, respectively, but not significantly different (p=0.2721), as seen in Table 2. PEP was the most common complication overall (6.7%).

**Table 2:** Complication rates in day cases compared to inpatient endoscopic retrograde cholangiopancreatography (ERCP)

Complications	Day Case (n=42)	Inpatient (n=92)
Post-ERCP pancreatitis	1 (2.4%)	8 (8.7%)
Bleed	0	1 (1.1%)
Cholecystitis	1 (2.4%)	0
Hospital-acquired pneumonia	0	1 (1.1%)
Overall	2 (4.8%)	10 (10.9%)

## Discussion

The majority of ERCPs in our study were performed as inpatient cases (n=92) compared to day cases (n=42), all of which were performed in one district general hospital in Northwest England. There was a huge disparity comparing same-day discharges between inpatient and day case patients. Only 3.3% of inpatient ERCPs were discharged the same day compared to 95.2% of day case ERCPs.

Following an inpatient ERCP, 88 patients were admitted overnight, of which 51% (n=45) were for post-ERCP next-day blood tests or observation despite being asymptomatic. None of these patients were diagnosed with complications, which suggests there is no benefit of performing investigations in asymptomatic patients. Furthermore, it resulted in a worse quality of stay for the patients, leading to a total of 63 additional nights a surgical bed was occupied and a total of 50 additional blood tests taken. This has the potential to cause delays in patients requiring emergency surgery due to bed pressures within the National Health Service (NHS), which could result in bad outcomes. There was one patient who was admitted for five days in hospital, awaiting LFTs to normalise despite showing no symptoms of complication. It has been reported in the literature by Silverman et al. that it is common for both LFTs and amylase to be deranged post ERCP asymptomatically [7]. 

In the day case ERCP cohort, no patients were admitted for asymptomatic investigations. This is likely due to the implementation of a more standardised post-ERCP protocol within the outpatient endoscopy unit with a post-procedural observation period followed by discharge. Conversely, this may indicate a lack of knowledge of ESGE guidelines amongst the general surgical department, who appear more cautious with same-day discharges without the reassurance of next-day blood tests or overnight observation. 

When comparing the complication rates of the two cohorts in our study, 14 patients who underwent an inpatient ERCP were admitted with symptoms suggestive of a complication, such as abdominal pain, vomiting or low blood pressure. Out of these 14 patients, eight were subsequently diagnosed with an ERCP-associated complication. In the day case cohort, only 2% (n=1) of patients were admitted with symptoms suggestive of a complication; this patient was subsequently diagnosed with cholecystitis. The increased complication rate seen for inpatient ERCP was not significant; however, it may be due to patient-specific risk factors which were not taken into account. Nearly half (43%) of patients admitted with symptoms suggestive of a complication following inpatient ERCP were not diagnosed with any ERCP-associated complication. This may be due to inadequate analgesia or missed diagnoses of complications. 

In the inpatient cohort, two patients were readmitted within seven days post discharge with ERCP-related complications: one with PEP and the other with hospital-acquired pneumonia. The patient re-admitted with PEP had already been admitted post ERCP due to a mild bleed; however, they unusually developed PEP a few days later following discharge. In the day case cohort, one patient was readmitted following discharge with PEP. The readmission rates were comparable between the two cohorts (2.2% and 2.4% for inpatient and day case, respectively), supporting current literature that day case ERCP does not increase the risk of developing a post-procedural complication within seven days [9]. 

The most common complication was PEP, which commonly presented initially with abdominal pain and/or vomiting. The incidence rates for PEP amongst the data we collected were similar to current literature, 8.7% and 2.4% for inpatients and day cases, respectively [4, 5]. There was no statistically significant difference in overall complication rates between day case and inpatient ERCP. Our findings support the safe running of the day case ERCP service. 

Limitations

There were a few limitations to our study. The data were collected as part of a single-centre retrospective audit with a total of only three ERCP operators. The majority of ERCPs were performed by a single operator. As the data were initially collected for an audit, no power calculation was performed for the sample size. All the data were collected from a single doctor, and the data were not independently validated, which could have led to potential observer bias and input errors. In addition, risk factors and co-morbidities were not compared between the two cohorts, which could have affected post-procedural complications. Further studies with larger sample sizes across multiple hospitals and a greater variety of clinicians would be beneficial to support our findings and determine if similar practices are repeated across England. A more equal distribution of day cases and inpatients in the sample size would also have been beneficial to develop more accurate conclusions when comparing the two cohorts.

Areas of further research

A retrospective study by Zhang et al. found dynamic fluctuations in leukocyte, neutrophil, and lymphocyte counts in patients developing PEP post ERCP [10]. These could be linked to the immunological response to infection and activation by the amylase enzyme in response to PEP development. However, PEP was not the only theory for such fluctuations, and it was suggested that it may be due to the body’s stress response to the procedure. This may further support the lack of relevance of reviewing immune markers to determine postoperative complications, especially in asymptomatic patients. Furthermore, fluctuations in immunological parameters were found not to be as sensitive as amylase.

A study by Geraghty et al. suggested the use of urinary trypsinogen as a biomarker to identify PEP, which remains the most common complication [11]. The biomarker was found to have a 99% negative predictive value and appears to be detected earlier than amylase. A dipstick can be taken by the nursing team, and if the patient is asymptomatic of abdominal pain, they can be discharged safely without the need for further investigations, reducing admission rates. This adds a further safety net for asymptomatic patients to be discharged the same day safely, and could have potentially picked up the two patients in our study who were re-admitted with PEP within seven days of discharge. 

## Conclusions

Our study supports current ESGE guidance that routine investigations following ERCP are not necessary in asymptomatic patients with no risk factors for complications. Over half of patients (51%) admitted following an inpatient ERCP could have been safely discharged the same day following a six-hour observation period without the need for further investigation. This resulted in 63 additional bed days over a six-month period. We have shown that the day case ERCP unit successfully implements a same-day discharge service, reducing the need for overnight admission, unnecessary investigations, and resources with no significant increase in complication rates. 

We propose that discharge planning for inpatients undergoing ERCP should begin prior to the procedure. The endoscopist or other experienced clinician should identify the patients who are not at high risk for complications and do not have ongoing medical management that requires admission. Such patients should be prepared for same-day discharge, given they remain asymptomatic following a six-hour observation period. Patients should be provided with adequate safety netting to be able to recognise early features of complications. This will lead to a reduction in patient length of stay, improve patient quality of stay and reduce costs to the hospital whilst maintaining patient safety. 
